# Craniofacial Morphology of Orthodontic Patients with and without Temporomandibular Disorders: A Cross-Sectional Study

**DOI:** 10.1155/2022/9344028

**Published:** 2022-03-22

**Authors:** Zhe-Bin Yan, Yi-Dan Wan, Chu-Qiao Xiao, Ya-Qi Li, Yu-Yao Zhang, Yang An, Xin Xiong

**Affiliations:** ^1^Department of Orthodontics, State Key Laboratory of Oral Disease, National Clinical Research Center for Oral Disease, West China Hospital of Stomatology, Sichuan University, Chengdu, China; ^2^Department of Nursing, West China Hospital of Stomatology, Sichuan University, Chengdu, China

## Abstract

**Purpose:**

We aimed to explore the relationship between temporomandibular disorders (TMDs) and craniofacial morphology in orthodontic patients.

**Methods:**

Altogether, 262 orthodontic patients were included and divided into two groups according to their Fonseca Anamnestic Index (FAI) scores: a no-TMD group (control group, FAI < 20) and a TMD group (FAI ≥ 20). Cephalometric parameters including cranial, maxillary, mandibular, and dental parameters were traced on cephalograms. Craniofacial morphology was compared between TMD and control groups, followed by subgroup analyses based on TMD severity, gender, age, and temporomandibular joint (TMJ) symptoms.

**Results:**

The prevalence of TMDs was 52.7% among included patients (138/262). The mean age of TMD patients was higher than that of the control group. No significant difference in gender distribution between the groups was observed. The most commonly reported FAI items were misaligned teeth, neck pain, and emotional tension. The Frankfort-mandibular plane angle (FMA) was larger in the TMD patients than in the control group, whereas no significant differences in other parameters were observed. Subgroup analysis based on TMD severity revealed that FMA and anterior facial height of moderate/severe TMD patients were significantly larger than those of mild or no-TMD patients. Among male patients, the anterior cranial base length was smaller, and the anterior facial height was larger in the TMD group. Among female patients, no significant differences in craniofacial morphology between the groups were observed. In juvenile patients, overjet and overbite were smaller in the TMD group. In adult patients, SNA, ANB, FMA, and gonial angle were larger in the TMD group. Within the TMD group, patients with TMJ pain or noises exhibited characteristic craniofacial features compared to patients without these symptoms.

**Conclusions:**

Orthodontic patients with TMDs have specific craniofacial morphology, suggesting a relationship between TMDs and particular craniofacial features in orthodontic patients.

## 1. Introduction

Temporomandibular disorders (TMDs) are a group of disorders involving pain and dysfunction of the temporomandibular joint (TMJ) and masticatory muscles [1]. The prevalence of TMDs is approximately 40% [2], with higher rates in individuals aged 20–40 years and in women [1, 3, 4]. The main symptoms of TMDs include pain in the face and TMJ area, the difficulty of mandibular movement, and TMJ noises during mandibular movement [1], which affects the quality of life for many patients. Currently, TMDs are considered multi-etiological disorders. The main risk factors include biological factors such as anatomical morphology, trauma, occlusion, and sex hormones alongside psychological factors such as anxiety and depression [5–8]. Since the etiology and pathogenesis of TMDs have not been fully clarified, more research on risk factors for TMDs is warranted.

Over the years, various methods of diagnosing TMDs have been proposed, including the Research Diagnostic Criteria for TMDs (RDC/TMD) [9] and the Diagnostic Criteria for TMDs (DC/TMD) [10], an improved version of the RDC/TMD. However, these instruments are procedurally complicated and require professional judgment, limiting their scope of application. A simplified and easier-to-use questionnaire is required to screen for TMDs in a larger population. A case in point is the Fonseca Anamnestic Index (FAI), which was proposed in 1994 and has shown high sensitivity in the diagnosis of TMDs [11–13]. The FAI consists of only ten questions pertaining to symptoms and risk factors related to TMDs. By calculating the total score, clinicians can diagnose the presence and severity of TMDs efficiently and at a low cost. In studies involving large sample populations and epidemiological surveys, the FAI would be superior to the RDC/TMD and DC/TMD for these reasons.

Nowadays, the clinical symptoms and signs of TMDs are relatively straightforward. Hence, other features of TMD patients, such as craniofacial morphology, are attracting growing academic attention. Patients with TMDs are reported to have specific craniofacial features such as a skeletal Class II profile, hyperdivergent growth pattern, and others [14, 15]. Some studies found differences in cervical posture and hyoid bone position between TMD patients and normal individuals, with an elevation of the hyoid bone and cervical lordosis observed in TMD patients [16, 17]. However, other studies have shown no differences in craniofacial morphology between TMD patients and individuals without TMDs [18–20]. The relationship between craniofacial morphology and TMDs remains controversial, especially in the orthodontic population. Therefore, the current study aimed to explore the relationship between the occurrence of TMDs and specific craniofacial morphological features among orthodontic patients. The null hypothesis was that there would be no relationship between them.

## 2. Materials and Methods

### 2.1. Subjects

The current observational cross-sectional study was performed at the Department of Orthodontics, West China Hospital of Stomatology, Sichuan University, during June 2021–August 2021. The study was approved by the Ethics Committee of West China School of Stomatology of Sichuan University (Ethics number: WCHSIRB-2020-418) and was conducted in accordance with the Declaration of Helsinki. All adult patients themselves and parents or legal guardians of patients aged under 18 years provided informed consent. The study was performed in compliance with the Strengthening the Reporting of Observational Studies in Epidemiology (STROBE) research guidelines [21].

Every orthodontic patient visiting the Department of Orthodontics for the first time was recruited consecutively and requested to fill out a questionnaire, including age, gender, medical history, and FAI scale. Angle's classification of malocclusion was determined by clinical examination. Lateral cephalograms were obtained in the Department of Imaging following routine procedures. The inclusion criteria were as follows: (1) patients seeking treatment in our department for the first time; (2) patients with qualified lateral cephalograms; and (3) patients aged 12 years or above. The exclusion criteria were as follows: (1) patients with orthodontic or orthognathic treatment history; (2) patients with maxillofacial trauma, cleft lip, or cleft palate; (3) patients with craniomaxillofacial deformities caused by systemic diseases such as rheumatoid arthritis; and (4) patients with mental disorders such as depression.

### 2.2. TMD Diagnosis and Severity Assessment

The Chinese version of the FAI [22] was utilized to assess whether a subject had TMD and to assess its severity. The scale consisted of ten questions, which in English are as follows: (1) Do you have difficulty opening your mouth wide? (2) Do you have difficulty moving your jaw to the sides? (3) Do you feel fatigued or muscle pain when you chew? (4) Do you have frequent headaches? (5) Do you have neck pain or a stiff neck? (6) Do you have ear pain or pain in the TMJ area? (7) Have you ever noticed any noise in your TMJ while chewing or opening your mouth? (8) Do you have any habits, such as clenching or grinding your teeth? (9) Do you feel that your teeth do not come together well? (10) Do you consider yourself a tense (nervous) person? Thus, the FAI allowed researchers to measure the degree of symptoms (questions 1–7) and risk factors (questions 8–10) related to TMDs of subjects. Each question had three possible answers, and each answer had a corresponding score (yes = 10, sometimes = 5, and no = 0). The severity of TMD of each patient could be assessed using the total FAI score (no-TMD = total score of 0–15, mild TMD = total score of 20–40, moderate TMD = total score of 45–65, and severe TMD = total score of 70–100) [11].

### 2.3. Cephalometric Analysis

**Figure 1:**
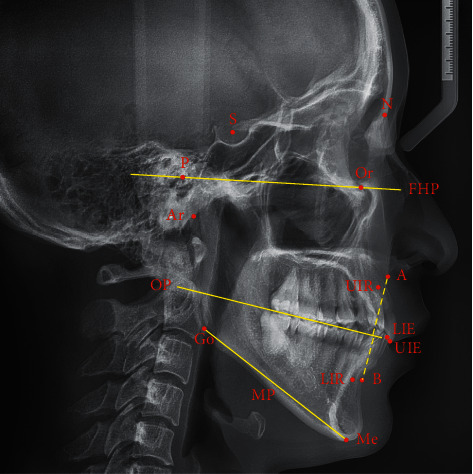
Cephalometric landmarks used in the study. N, Nasion; S, Sella; P, Porion; Or, Orbitale; Ar, Articulare; A, Subspinale; UIR, Upper incisor root; UIE, Upper incisor edge; LIR, Lower incisor root; LIE, Lower incisor edge; B, Supramentale; Me, Menton; Go, Gonion; FHP, Frankfort horizontal plane; OP, Occlusal plane; MP, Mandibular plane.

The lateral cephalograms of all patients were obtained by the same radiologist. Patients were required to maintain the natural head position [23] with the mandible in the maximum intercuspal position. After obtaining the lateral cephalograms, Uceph software (version 780, Yacent, Chengdu, Sichuan, China) was used for cephalometric analysis. The cephalometric landmarks used in the study are shown in Figure 1.

The eighteen cephalometric parameters used in the study are presented as follows. Cranial parameters: (1) Saddle angle (N-S-Ar angle); (2) Articular angle (S-Ar-Go angle); (3) Anterior cranial base length (S-N distance); (4) Posterior cranial base length (S-Ar distance). Maxillary and mandibular parameters: (1) SNA (S-N-A angle); (2) SNB (S-N-B angle); (3) ANB (A-N-B angle); (4) FMA (Frankfort horizontal plane-mandibular plane angle); (5) Gonial angle (Ar-Go-Me angle); (6) Ramus height (Ar-Go distance); (7) Mandibular Body length (Go-Me distance). Dental parameters: (1) Interincisal angle (U1E-U1R-L1E-L1R angle); (2) Cant of occlusal plane (Frankfort horizontal plane-occlusal plane angle); (3) Overjet (U1E-L1E horizontal distance); (4) Overbite (U1E-L1E vertical distance). Other parameters: (1) Anterior facial height (N-Me distance); (2) Posterior facial height (S-Go distance); (3) Wits appraisal (A' (the intersection between the perpendicular of occlusal plane through A and occlusal plane)-B' (the intersection between the perpendicular of occlusal plane through B and occlusal plane) distance).

Two researchers blinded to the patient details performed the measurements. Inter-observer and intra-observer reliability were tested according to the method described by Xiong et al. [24] to ensure the accuracy of the measurements. For inter-observer reliability, 20 lateral cephalograms were randomly selected for initial measurement by the researchers before the formal measurement, and the repeat measurement was performed 2 weeks later. The intra-class correlation coefficient (ICC) was used to test the repeatability of the results from the two measurements. If ICC was ≥ 0.75, formal measurement was performed. If ICC was < 0.75, 20 cephalograms were randomly selected for repeatability testing again. Formal measurement was not completed until an ICC value of ≥ 0.75 was obtained.

### 2.4. Statistical Analysis

The demographic, occlusal, and craniofacial morphological characteristics were compared between the control group (FAI < 20) and TMD group (FAI ≥ 20), followed by subgroup analyses based on TMD severity, gender, and age. According to the results of item 6 in the FAI scale, the TMD group was divided into subgroups of patients with TMJ pain (item 6 score = 5 or 10) and without TMJ pain (item 6 score = 0). According to the results of item 7, the TMD group was divided into subgroups of patients with TMJ noises (item 7 score = 5 or 10) and without TMJ noises (item 7 score = 0). The demographic and occlusal characteristics as well as cephalometric parameters were compared between patients with and without TMJ pain, as well as between patients with and without TMJ noises.

All data were analyzed using IBM SPSS Statistics (version 20.0, IBM Corp., Armonk, NY, USA). Quantitative data were expressed as mean and standard deviation (SD), and qualitative data were expressed as quantity and frequency. The Shapiro-Wilk test was used to analyze the normality of data distribution. To compare the differences in ages and cephalometric parameters between the groups, an independent samples *t*-test or one-way analysis of variance was used when the data showed a normal distribution. Mann-Whitney *U* test or Kruskal-Wallis *H*-test was used when the data did not show a normal distribution. A chi-squared test was used to analyze the differences in sex distribution and occlusal characteristics between the groups. The test level was *α* = 0.05, and *P* < 0.05 was taken to indicate statistically significant differences.

## 3. Results

### 3.1. Demographic and Occlusal Characteristics

Altogether, 262 orthodontic patients were included in this study. The mean age was 21.20 ± 7.11 years, and the proportion of women was 65.3%. According to the FAI classification criteria, patients were divided into two groups: a no-TMD group (control group, 124 patients [47.3%]) and a TMD group (138 patients [52.7%]). The mean age of the TMD group was significantly higher than that of the no-TMD group (*P* < 0.001). However, no significant difference in gender distribution between the groups was observed (*P*=0.307). The proportions of females were higher than those of males in both groups. The proportion of patients with Angle Class III in the TMD group was significantly higher than that in the no-TMD group (*P*=0.019, Table 1).

### 3.2. FAI Scale Survey

**Figure 2:**
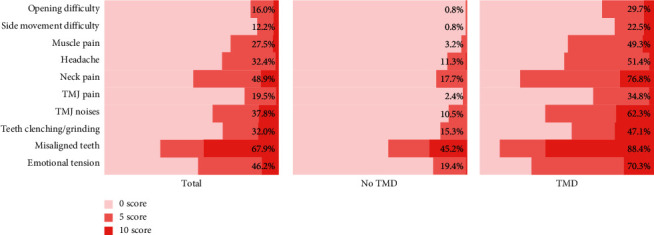
Results of the Fonseca anamnestic index (FAI) scale survey. Left panel, all included patients; center panel, patients with no TMDs; and right panel, patients with TMDs. Each bar shows the answer distribution of an item on the FAI survey. The percentage in each row represents the proportion of patients with a score of 5 or 10, indicating the prevalence rate of the TMD-related symptom or risk factor reflected by every question. TMJ: temporomandibular joint, TMD: temporomandibular disorder.

Among the included patients, the most commonly reported TMD symptoms were neck pain (48.9%) and TMJ noises (37.8%), followed by headache (32.4%) and muscle pain (27.5%). The frequencies of all three TMD risk factors were high, with the prevalence of misaligned teeth (67.9%) the highest. The frequencies of various clinical manifestations were low in the no-TMD group, with only the presence of misaligned teeth (45.2%) exhibiting a frequency above 20%. However, a variety of widespread symptoms and risk factors were present in the TMD group. Misaligned teeth (88.4%) and neck pain (76.8%) were observed in most of the patients (Figure 2).

### 3.3. Relationship between TMDs and Craniofacial Morphology

Table 2 compares craniofacial morphological parameters between the no-TMD group and the TMD group. A significant difference in FMA between the groups was observed. The average FMA in the TMD group was larger than that in the control group (*P* = 0.039).

After stratifying the TMD patients according to severity, the demographic, occlusal, and craniofacial morphological characteristics were compared among patients without TMDs, with mild TMDs, and with moderate/severe TMDs (Table 3). Patients with mild and moderate/severe TMDs were older on average than those without TMDs (*P* < 0.001). No differences were observed in gender or Angle's classification distribution among the three groups (*P* > 0.05). Significant differences were observed in FMA and anterior facial height among the three groups. FMA and anterior facial height were significantly larger in patients with more severe TMDs (*P* < 0.05).

After stratifying the patients by sex, no difference was observed in age between the no-TMD group and the TMD group among male patients (Table 4, *P*=0.120). However, female patients in the TMD group were significantly older on average than those in the control group (Supplement Table 1, *P*=0.001). No significant differences in occlusal characteristics were noted between the control and TMD groups of the same sex (*P* > 0.05). In male patients, the anterior cranial base length of the TMD group was shorter than that of the control group, while the anterior facial height of the TMD group was longer (*P* < 0.05). Nevertheless, in female patients, no significant differences were observed in craniofacial morphology between the TMD and control groups (*P* > 0.05).

After stratifying the patients by age (stratification boundary: 18 years), both in juvenile (aged under 18 years) and adult patients (aged 18 years or above), no statistical differences existed in gender or Angle's classification distribution between the groups (Table 5 and Supplement Table 2, *P* > 0.05). Among juveniles, TMD patients were older than those without TMDs (*P*=0.001). Among adults, no significant difference was observed in age between the groups (*P*=0.917). Among juvenile patients, the overjet and overbite were significantly smaller in TMD patients than in the control group (*P* < 0.05). Among adults, SNA, ANB, FMA, and gonial angle were significantly larger in TMD patients than in the control group (*P* < 0.05).

Within the TMD group, the proportion of males with TMJ pain was significantly higher than that of males without TMJ pain (Table 6, *P*=0.010). No significant differences were observed in age or occlusal characteristics between patients with and without TMJ pain (*P* > 0.05). Within the TMD group, the posterior cranial base length and Wits appraisal value of patients with TMJ pain were larger than those of patients without TMJ pain (*P* < 0.05).

No significant differences were noticed in demographic or occlusal characteristics between TMD patients with and without TMJ noises (Table 7, *P* > 0.05). The gonial angle of patients with TMJ noises was significantly greater than that of patients without TMJ noises (*P*=0.047).

## 4. Discussion

In the present study, we observed differences in craniofacial morphology between individuals with TMDs and controls without TMDs among a population of orthodontic patients. Especially in male patients, the anterior cranial base length in the TMD group was shorter on average than in the control group, while the anterior facial height of the TMD group was larger; in adult patients, the SNA, ANB, FMA, and gonial angle were larger in TMD patients than in controls. Therefore, the null hypothesis—that there would be no relationship between specific craniofacial morphology and TMDs in this population—was rejected.

The mean age of TMD patients was significantly higher than that of patients without TMDs, which was consistent with the results reported by Yap et al. [25]. This may be attributed to the high incidence of TMD symptoms in populations of middle age [26] or the pathological changes in the TMJ and masticatory muscles, which are easily affected by internal and external environmental factors after the period of growth. However, since only orthodontic patients were included in the present study, all patients (with and without TMDs) were younger than the population studied by Yap et al. [25]. No difference was observed in gender distribution between the TMD group and the control group in the present study. The proportions of women in the study-enrolled group overall, the control group, and the TMD group were similar and higher than those of men, in agreement with the findings reported by Almasan et al. [27]. Biological factors such as genes, hormones, pain perception, psychosocial factors, environmental factors, and others may be associated with the higher prevalence of TMDs in women [4]. The high level of estrogen in women's bodies may be one reason for the higher incidence of TMDs. However, there is no consensus about the relationship between estrogen level and prevalence of TMDs [28]. It is suggested to further explore the specific role of estrogen in the occurrence and development of TMDs. Additionally, the percentage of patients categorized as Angle Class III in the TMD group was significantly higher than in the control group. However, no statistical differences in Angle's classification distribution were noted in subgroup analyses. These results may indicate that the relationship between occlusal factors and the occurrence of TMDs is weak. This implies that other factors such as social, psychological, and environmental influences should be emphasized in the etiology of TMDs [29].

Before stratification, the FMA of TMD patients was significantly larger than that of patients without TMDs. Subgroup analysis based on the severity of TMDs showed that patients with more severe TMDs had greater FMA. Thus, the facial shape of TMD patients tended towards a hyperdivergent growth pattern. Similar trends have been reported by Hwang et al. in their work [15]. After stratification, the anterior facial height of patients with moderate/severe TMDs was significantly greater than that of patients with no or mild TMDs. No such difference was observed before stratification. Similar results were obtained in the experiment by Ramirez-Caro and Espinosa de Santillana [20], who reported that adolescent patients with TMDs exhibited a greater lower facial height. In contrast, Joy et al. [30] found that patients with more severe TMDs exhibited decreased anterior and posterior facial heights. This contradiction between Joy et al. and our results could be attributed to the fact that the former study included patients with a reduced vertical dimension of occlusion (VDO). VDO can be measured using the Shimbashi number—the distance between the cementoenamel junctions of the upper and lower incisors [30]. Decreased VDO may be one of the risk factors for TMDs. It also affects the shape of the face directly, especially that of the lower face, which might explain the results in the study mentioned above. Therefore, we postulate that the orthodontic patients with TMDs exhibit trends towards a hyperdivergent growth and characteristically long faces.

Subgroup analysis based on gender showed that among male patients, the anterior facial height of the TMD group was larger, which was similar to the results before stratification. The anterior cranial base length of the TMD group was shorter than that of the control group. Nevertheless, no significant differences were observed in any of the cephalometric parameters between the TMD and control groups in female patients. Kwon et al. [31] found that in both men and women, patients with TMJ disc displacement showed different dentofacial morphology compared with the normal population. In a study by Chen et al. [32] involving female patients with skeletal Class II deformity, the craniofacial morphology of osteoarthrosis patients was different from that of the control group. In contrast to these earlier findings, our results did not note any special craniofacial features of female patients with TMDs. This might be partially attributed to the insufficient sample size. In the present study, although no significant differences in any parameters between TMD and control groups were observed among female patients, the *P*-values obtained from statistical analyses of the anterior facial height, anterior cranial base length, and other parameters were still close to the test level. Significant differences might be observed in these parameters if the sample size were expanded. Moreover, images were analyzed to diagnose TMDs in these previous studies, while a symptom-based scale was utilized in our study. This suggests that objective imaging data can accurately diagnose TMDs and may be more conducive to finding the specific characteristics of craniofacial morphology in female patients with TMDs.

Subgroup analysis based on age revealed statistically significant differences in overjet and overbite between the TMD and control groups among juvenile patients. Venere et al. [18] found that the proportion of deep bites in patients with craniomandibular disorders was higher than in their control group, but the difference was not statistically significant. Pereira et al. [19] found that larger overjet was associated with higher Dysfunction Index scores in patients with TMDs. Sonnesen et al. [33] found that larger overjet and smaller overbite were associated with a high prevalence of TMD-related symptoms. However, these studies could not confirm significant differences in craniofacial morphology between adolescents and children with TMDs and those without. In contrast, significant differences were observed in SNA, ANB, FMA, and gonial angle between the groups in adult patients. In the current study, adult patients with TMDs exhibited more protruding maxilla, steeper mandible, and larger gonial angle, which reinforced the conclusions of previous studies [15, 34]. The relationships between TMDs and craniofacial morphology were inconsistent among different age groups. This may be explained by the fact that children and adolescents are still in the growth phase, with their bone structures still affected by age and nutrition. Thus, the craniofacial morphology of these TMD patients may not exhibit unique characteristics. In comparison to youngsters, the craniofacial bones in adults, who have passed the peak of growth, tend to be more stable. The occurrence and development of TMDs may be associated with significant remodeling of bones, which may result in more apparent differences in craniofacial morphological parameters between the TMD and control groups.

In the TMD group, both the posterior cranial base length and Wits appraisal value of patients with TMJ pain were greater than controls, suggesting that patients with a longer posterior cranial base and Angle Class II malocclusion may have overloaded TMJs and be more prone to pain. The gonial angle of patients with TMJ noises was larger than those without, which may indicate its relationship with pathophysiological changes in the TMJ, resulting in TMJ complaints. Colonna et al. [34] found that patients with TMJ pain had a larger gonial angle. Dibbets and van der Weele [35] also found that orthodontic patients with TMJ clicking or crepitations had smaller maxilla, mandible, and cranial base lengths. These findings suggest that patients with local TMJ symptoms exhibit characteristic craniofacial morphology, which is in line with our results. The occurrence of some potential TMJ symptoms may be predicted using these unique parameters, but more studies are required to determine the specific craniofacial morphological characteristics among these patients.

Several previous studies were carried out with limited sample sizes and mainly focused on female or adult patients. The sample size in the present study was relatively larger, and subgroup analyses based on sex, age, and TMJ symptoms were conducted. The characteristic craniofacial features of TMD patients were mainly discovered in men and adults, while in women, no differences were observed in cephalometric parameters between control and TMD groups. In addition, some craniofacial morphological features were observed in patients with TMJ pain or noises within the TMD group. These findings suggest that craniofacial morphology could be used as a screening and diagnostic tool for TMDs and indicate a new direction for applying lateral cephalograms in the future [15]. However, because several factors such as social background, periodontal, and mucosal status were not controlled in this study, further research is required to explore the specific craniofacial morphology of TMD patients and verify this conjecture.

The present study has some limitations. Initially, the duration of TMD symptoms was not considered. TMD patients who have long experienced symptoms might have undergone evident bone remodeling and thus may exhibit unique craniofacial morphology. Failure to stratify TMD patients according to the duration of the illness might miss some parameters with significant differences. Furthermore, due to the large sample population this study recruited, the FAI scale was utilized to diagnose TMDs due to its simplicity and convenience. However, because of its low specificity [13], some false-positive TMD patients might have been included. Therefore, in future studies, both FAI and DC/TMD could be used together to screen and diagnose TMDs. Moreover, the cone beam computed tomography and magnetic resonance imaging of the TMJ, which are helpful for the diagnosis of TMD subtypes such as TMJ degenerative joint diseases and disc displacement, should be evaluated. In addition, the present study included only orthodontic patients, some of whom were so young that they may have not developed TMDs yet, but could in the future. This may lead to a different prevalence rate of TMDs in this study compared to others, making it difficult to detect special craniofacial morphology of TMD patients. Thus, further studies involving other populations, such as patients from the Department of TMJ, are needed.

## 5. Conclusions

We observed a significant difference in FMA between individuals with and without TMDs among the studied population of orthodontic patients. In male patients and adult patients, significant differences in craniofacial morphology between the control and TMD groups were present in more parameters, including anterior facial height, anterior cranial base length, ANB, and gonial angle. In female patients and juvenile patients, the differences in craniofacial morphology between the groups were not significant. Orthodontic patients with TMDs have a particular type of craniofacial morphology, especially in male and adult patients. A specific relationship between TMDs and craniofacial features exists. In patients with TMDs, differences in posterior cranial base length, Wits appraisal, and gonial angle were observed between patients with and without TMJ pain or noises, suggesting possible relationships between TMJ symptoms and craniofacial morphology in TMD patients.

## Figures and Tables

**Table 1:** Demographic and occlusal characteristics of patients with and without TMDs.

		Total	No TMD	TMD	*P*-value
Number	(*n* (%))	262 (100.0)	124 (47.3)	138 (52.7)	

Age	(years, mean ± SD)	21.20 ± 7.11	19.46 ± 7.44	22.76 ± 6.44	<0.001^*∗*^

Age stratification	<18 years (*n* (%))	90 (34.4)	60 (48.4)	30 (21.7)	
	≥18 years (*n* (%))	172 (65.6)	64 (51.6)	108 (78.3)	

Gender	Male (*n* (%))	91 (34.7)	47 (37.9)	44 (31.9)	0.307
	Female (*n* (%))	171 (65.3)	77 (62.1)	94 (68.1)	

Angle's classification	Angle class I (*n* (%))	103 (39.3)	51 (41.2)	52 (37.7)	0.019^*∗*^
	Angle class II (*n* (%))	97 (37.0)	53 (42.7)	44 (31.9)	
	Angle class III (*n* (%))	62 (23.7)	20 (16.1)	42 (30.4)	

Independent samples *t*-test and chi-squared test were used. ^*∗*^*P* < 0.05. TMD: temporomandibular disorder, SD: standard deviation.

**Table 2:** Comparison of craniofacial morphological parameters between patients with and without TMDs.

	Total	No TMD	TMD	*P*-value
	262 (100.0%)	124 (47.3%)	138 (52.7%)	
	Mean ± SD	Mean ± SD	Mean ± SD	
Saddle angle (°)	122.91 ± 5.10	123.26 ± 4.41	122.59 ± 5.64	0.378
Articular angle (°)	151.47 ± 6.83	151.04 ± 6.31	151.86 ± 7.26	0.330
Anterior cranial base length (mm)	64.18 ± 3.69	64.38 ± 3.87	63.99 ± 3.53	0.657
Posterior cranial base length (mm)	34.67 ± 3.50	34.71 ± 3.24	34.64 ± 3.73	0.873
SNA (°)	82.04 ± 3.27	81.73 ± 3.23	82.31 ± 3.30	0.156
SNB (°)	79.00 ± 4.24	79.02 ± 3.96	78.98 ± 4.49	0.953
ANB (°)	3.04 ± 3.54	2.72 ± 3.46	3.32 ± 3.59	0.176
FMA (°)	24.08 ± 6.77	23.17 ± 5.95	24.89 ± 7.35	0.039*∗*
Gonial angle (°)	118.20 ± 7.60	117.49 ± 6.57	118.83 ± 8.40	0.150
Ramus height (mm)	47.17 ± 5.96	47.20 ± 5.74	47.15 ± 6.17	0.934
Mandibular body length (mm)	70.73 ± 5.24	70.70 ± 5.22	70.76 ± 5.27	0.919
Interincisal angle (°)	124.73 ± 13.99	124.34 ± 13.28	125.08 ± 14.64	0.993
Cant of occlusal plane (°)	7.06 ± 4.78	6.65 ± 4.92	7.44 ± 4.63	0.180
Overjet (mm)	3.75 ± 3.46	3.97 ± 3.42	3.55 ± 3.50	0.227
Overbite (mm)	2.68 ± 2.20	2.83 ± 2.03	2.54 ± 2.34	0.244
Anterior facial height (mm)	116.90 ± 8.29	116.07 ± 7.84	117.65 ± 8.63	0.124
Posterior facial height (mm)	79.23 ± 7.80	79.24 ± 7.37	79.22 ± 8.20	0.982
Wits appraisal (mm)	−0.05 ± 5.15	−0.14 ± 4.63	0.03 ± 5.59	0.473

Independent samples *t*-test and Mann–Whitney *U*-test were used. ^*∗*^*P* < 0.05. TMD: temporomandibular disorder, SD: standard deviation.

**Table 3:** Comparison of demographic, occlusal, and craniofacial morphological characteristics among patients with no, mild, and moderate/severe TMDs.

		No TMD	Mild TMD	Moderate/severe TMD	*P* value
		124 (47.3%)	100 (38.2%)	38 (14.5%)	
Age (years, mean ± SD)		19.46 ± 7.44^a^	22.57 ± 6.73^b^	23.24 ± 5.66^b^	<0.001^∗^
Gender	Male (*n* (%))	47 (37.9)	30 (30.0)	14 (36.8)	0.447
	Female (*n* (%))	77 (62.1)	70 (70.0)	24 (63.2)	
Angle's classification	Angle class I (*n* (%))	51 (41.2)	39 (39.0)	13 (34.2)	0.078
	Angle class II (*n* (%))	53 (42.7)	32 (32.0)	12 (31.6)	
	Angle class III (*n* (%))	20 (16.1)	29 (29.0)	13 (34.2)	
Craniofacial morphological parameters (mean ± SD)					
Saddle angle (°)		123.26 ± 4.41	123.05 ± 5.09	121.38 ± 6.82	0.566
Articular angle (°)		151.04 ± 6.31	151.53 ± 7.62	152.74 ± 6.20	0.406
Anterior cranial base length (mm)		64.38 ± 3.87	64.06 ± 3.50	63.80 ± 3.64	0.894
Posterior cranial base length (mm)		34.71 ± 3.24	34.45 ± 3.61	35.13 ± 4.05	0.594
SNA (°)		81.73 ± 3.23	82.12 ± 3.22	82.82 ± 3.50	0.207
SNB (°)		79.02 ± 3.96	78.88 ± 4.20	79.27 ± 5.21	0.886
ANB (°)		2.72 ± 3.46	3.24 ± 3.32	3.55 ± 4.26	0.334
FMA (°)		23.17 ± 5.95^a^	24.29 ± 7.28^ab^	26.47 ± 7.39^b^	0.028^*∗*^
Gonial angle (°)		117.49 ± 6.57	118.15 ± 8.37	120.63 ± 8.32	0.083
Ramus height (mm)		47.20 ± 5.74	47.10 ± 6.16	47.28 ± 6.28	0.662
Mandibular Body length (mm)		70.70 ± 5.22	70.77 ± 5.11	70.75 ± 5.73	0.995
Interincisal angle (°)		124.3 4± 13.28	126.63 ± 14.56	121.00 ± 14.24	0.140
Cant of occlusal plane (°)		6.65 ± 4.92	7.62 ± 4.69	6.97 ± 4.49	0.315
Overjet (mm)		3.97 ± 3.42	3.36 ± 3.44	4.04 ± 3.65	0.346
Overbite (mm)		2.83 ± 2.03	2.73 ± 2.32	2.05 ± 2.35	0.200
Anterior facial height (mm)		116.07 ± 7.84^a^	116.72 ± 8.38^a^	120.10 ± 8.92^b^	0.031^*∗*^
Posterior facial height (mm)		79.24 ± 7.37	78.92 ± 8.12	80.01 ± 8.48	0.434
Wits appraisal (mm)		−0.14 ± 4.63	−0.09 ± 5.21	0.35 ± 6.56	0.661

One-way analysis of variance, Kruskal–Wallis *H*-test, and chi-squared test were used. ^a, b^The same letters indicate no statistically significant difference, while different letters indicate a statistically significant difference between the groups. ^∗^*P* < 0.05 TMD: temporomandibular disorder, SD: standard deviation.

**Table 4:** Comparison of demographic, occlusal, and craniofacial morphological characteristics between male patients with and without TMDs.

		Males with no TMD	Males with TMD	*P* value
		47 (51.6%)	44 (48.4%)	
Age (years, mean ± SD)		19.06 ± 6.37	21.13 ± 6.20	0.120
Angle's classification	Angle class I (*n* (%))	20 (42.6)	16 (36.4)	0.165
	Angle class II (*n* (%))	19 (40.4)	13 (29.5)	
	Angle class III (*n* (%))	8 (17.0)	15 (34.1)	
Craniofacial morphological parameters (mean ± SD)				
Saddle angle (°)		123.54 ± 3.74	122.58 ± 6.28	0.924
Articular angle (°)		149.26 ± 5.85	151.51 ± 7.46	0.111
Anterior cranial base length (mm)		67.17 ± 3.79	65.50 ± 3.70	0.035^*∗*^
Posterior cranial base length (mm)		36.57 ± 2.82	37.52 ± 3.29	0.069
SNA (°)		81.85 ± 2.71	82.63 ± 3.27	0.116
SNB (°)		79.67 ± 3.91	79.64 ± 4.76	0.973
ANB (°)		2.18 ± 3.43	3.00 ± 4.27	0.189
FMA (°)		21.09 ± 6.23	23.65 ± 7.59	0.204
Gonial angle (°)		116.57 ± 6.80	117.31 ± 9.16	0.662
Ramus height (mm)		51.20 ± 5.66	51.43 ± 5.58	0.848
Mandibular body length (mm)		73.17 ± 5.69	73.29 ± 5.59	0.915
Interincisal angle (°)		122.73 ± 14.24	123.78 ± 14.07	0.724
Cant of occlusal plane (°)		5.54 ± 5.28	7.59 ± 4.73	0.056
Overjet (mm)		3.77 ± 4.04	3.31 ± 4.52	0.413
Overbite (mm)		3.00 ± 1.99	2.49 ± 2.47	0.275
Anterior facial height (mm)		120.00 ± 7.11	123.49 ± 8.69	0.038^*∗*^
Posterior facial height (mm)		84.60 ± 6.75	86.09 ± 7.06	0.335
Wits appraisal (mm)		−0.21 ± 4.67	−0.44 ± 7.03	0.715

Independent samples *t*-test and Mann-Whitney *U*-test were used. ^∗^*P* < 0.05. TMD: temporomandibular disorder, SD: standard deviation.

**Table 5:** Comparison of demographic, occlusal, and craniofacial morphological characteristics between adult patients (aged 18 years or above) with and without TMDs.

		Adults with no TMD	Adults with TMD	*P* value
		64 (37.2%)	108 (62.8%)	
Age (years, mean ± SD)		24.74 ± 6.81	24.84 ± 5.66	0.917
Gender	Male (*n* (%))	26 (40.6)	31 (28.7)	0.108
	Female (*n* (%))	38 (59.4)	77 (71.3)	
Angle's classification	Angle class I (*n* (%))	28 (43.7)	41 (38.0)	0.189
	Angle class II (*n* (%))	25 (39.1)	35 (32.4)	
	Angle class III (*n* (%))	11 (17.2)	32 (29.6)	
Craniofacial morphological parameters (mean ± SD)				
Saddle angle (°)		123.74 ± 4.52	122.51 ± 5.82	0.178
Articular angle (°)		150.98 ± 6.54	152.27 ± 7.10	0.237
Anterior cranial base length (mm)		65.20 ± 3.77	64.34 ± 3.54	0.278
Posterior cranial base length (mm)		34.98 ± 3.54	34.71 ± 3.78	0.645
SNA (°)		81.24 ± 3.23	82.28 ± 3.35	0.048^*∗*^
SNB (°)		79.23 ± 3.98	78.95 ± 4.59	0.689
ANB (°)		2.02 ± 3.57	3.33 ± 3.77	0.023^*∗*^
FMA (°)		21.95 ± 6.43	24.68 ± 7.33	0.015^*∗*^
Gonial angle (°)		116.00 ± 6.14	118.24 ± 8.09	0.042^*∗*^
Ramus height (mm)		49.24 ± 5.83	47.67 ± 6.29	0.107
Mandibular Body length (mm)		72.09 ± 5.00	71.13 ± 5.13	0.233
Interincisal angle (°)		124.72 ± 12.44	125.76 ± 15.31	0.646
Cant of occlusal plane (°)		5.91 ± 5.39	7.24 ± 4.68	0.093
Overjet (mm)		3.09 ± 3.03	3.52 ± 3.59	0.218
Overbite (mm)		2.47 ± 2.17	2.60 ± 2.42	0.730
Anterior facial height (mm)		117.60 ± 7.51	118.21 ± 8.52	0.635
Posterior facial height (mm)		81.48 ± 7.66	79.88 ± 8.19	0.207
Wits appraisal (mm)		−0.60 ± 4.30	0.22 ± 5.76	0.173

Independent samples *t*-test, Mann-Whitney *U*-test, and chi-squared test were used. ^∗^*P* < 0.05. TMD: temporomandibular disorder, SD: standard deviation.

**Table 6:** Comparison of demographic, occlusal, and craniofacial morphological characteristics between TMD patients with and without TMJ pain.

		TMD without TMJ pain	TMD with TMJ pain	*P* value
		90 (65.2%)	48 (34.8%)	
Age (years, mean ± SD)		23.29 ± 6.80	21.76 ± 5.66	0.187
Gender	Male (*n* (%))	22 (24.4)	22 (45.8)	0.010^*∗*^
	Female (*n* (%))	68 (75.6)	26 (54.2)	
Angle's classification	Angle class I (*n* (%))	37 (41.1)	15 (31.2)	0.193
	Angle class II (*n* (%))	24 (26.7)	20 (41.7)	
	Angle class III (*n* (%))	29 (32.2)	13 (27.1)	
Craniofacial morphological parameters (mean ± SD)				
Saddle angle (°)		122.46 ± 5.05	122.83 ± 6.67	0.324
Articular angle (°)		152.12 ± 7.52	151.37 ± 6.79	0.566
Anterior cranial base length (mm)		63.86 ± 3.55	64.23 ± 3.50	0.278
Posterior cranial base length (mm)		34.00 ± 3.68	35.84 ± 3.56	0.005^*∗*^
SNA (°)		82.29 ± 3.26	82.35 ± 3.42	0.914
SNB (°)		79.20 ± 4.41	78.58 ± 4.64	0.441
ANB (°)		3.09 ± 3.87	3.77 ± 2.97	0.291
FMA (°)		24.81 ± 7.89	25.04 ± 6.30	0.852
Gonial angle (°)		118.77 ± 8.90	118.94 ± 7.44	0.909
Ramus height (mm)		47.13 ± 6.20	47.18 ± 6.19	0.969
Mandibular Body length (mm)		70.89 ± 5.32	70.53 ± 5.21	0.705
Interincisal angle (°)		126.25 ± 14.10	122.89 ± 15.51	0.160
Cant of occlusal plane (°)		7.77 ± 4.79	6.83 ± 4.31	0.259
Overjet (mm)		3.16 ± 3.69	4.27 ± 3.02	0.498
Overbite (mm)		2.42 ± 2.19	2.76 ± 2.60	0.417
Anterior facial height (mm)		117.12 ± 8.74	118.65 ± 8.43	0.323
Posterior facial height (mm)		78.62 ± 8.19	80.34 ± 8.19	0.244
Wits appraisal (mm)		−0.70 ± 5.72	1.41 ± 5.12	0.027^*∗*^

Independent samples *t*-test, Mann–Whitney *U*-test, and chi-squared test were used. ^∗^*P* < 0.05. TMD: temporomandibular disorder, TMJ: temporomandibular joint, SD: standard deviation.

**Table 7:** Comparison of demographic, occlusal, and craniofacial morphological characteristics between TMD patients with and without TMJ noises.

			TMD without TMJ noises	TMD with TMJ noises	*P*-value
			52 (37.7%)	86 (62.3%)	
Age (years, mean ± SD)			23.37 ± 7.20	22.38 ± 5.95	0.385
Gender	Male (*n* (%))		18 (34.6)	26 (30.2)	0.592
	Female (*n* (%))		34 (65.4)	60 (69.8)	
Angle's classification	Angle class I (*n* (%))		25 (48.1)	27 (31.4)	0.128
	Angle class II (*n* (%))		15 (28.8)	29 (33.7)	
	Angle class III (*n* (%))		12 (23.1)	30 (34.9)	
Craniofacial morphological parameters (mean ± SD)					
Saddle angle (°)		123.21 ± 5.24		122.22 ± 5.86	0.322
Articular angle (°)		151.41 ± 7.10		152.14 ± 7.38	0.572
Anterior cranial base length (mm)		64.28 ± 3.34		63.82 ± 3.64	0.660
Posterior cranial base length (mm)		34.72 ± 3.33		34.59 ± 3.97	0.839
SNA (°)		82.50 ± 3.21		82.19 ± 3.37	0.598
SNB (°)		78.95 ± 3.58		79.01 ± 4.98	0.941
ANB (°)		3.55 ± 2.88		3.19 ± 3.96	0.874
FMA (°)		23.48 ± 7.08		25.75 ± 7.42	0.080
Gonial angle (°)		117.01 ± 8.34		119.93 ± 8.28	0.047^*∗*^
Ramus height (mm)		47.68 ± 5.55		46.83 ± 6.53	0.438
Mandibular body length (mm)		70.98 ± 5.00		70.63 ± 5.45	0.623
Interincisal angle (°)		126.89 ± 14.52		123.99 ± 14.69	0.160
Cant of occlusal plane (°)		7.42 ± 4.69		7.45 ± 4.62	0.966
Overjet (mm)		3.40 ± 2.91		3.64 ± 3.82	0.363
Overbite (mm)		2.91 ± 2.30		2.32 ± 2.34	0.316
Anterior facial height (mm)		116.44 ± 8.84		118.38 ± 8.47	0.201
Posterior facial height (mm)		79.75 ± 7.76		78.90 ± 8.49	0.738
Wits appraisal (mm)		0.51 ± 4.33		−0.26 ± 6.24	0.816

Independent samples *t*-test, Mann-Whitney *U*-test, and chi-squared test were used. ^*∗*^*P* < 0.05. TMD: temporomandibular disorder, TMJ: temporomandibular joint, SD: standard deviation.

## Data Availability

The data used to support the findings of this study are available from the corresponding author upon request.

## Supplementary Materials

Supplementary Materials
*Supplementary 1.* Supplement Table 1: Comparison of demographic, occlusal, and craniofacial morphological characteristics between female patients with and without TMDs. *Supplementary 2*. Supplement Table 2: Comparison of demographic, occlusal, and craniofacial morphological characteristics between juvenile patients (aged under 18 years) with and without TMDs.
