# Supplementary material for: Craniofacial Morphology of Orthodontic Patients with and without Temporomandibular Disorders: A Cross-Sectional Study
Source: Pain Res Manag. 2022 Mar 22;2022:9344028. doi: 10.1155/2022/9344028 (PMC8964235; doi:10.1155/2022/9344028)
Supplement: Supplementary Materials — Supplementary 1. Supplement Table 1: Comparison of demographic, occlusal, and craniofacial morphological characteristics between female patients with and without TMDs. Supplementary 2. Supplement Table 2: Comparison of demographic, occlusal, and craniofacial morphological characteristics between juvenile patients (aged under 18 years) with and without TMDs. [file 9344028.f1.docx]

**Supplement Table 1**. Comparison of demographic, occlusal, and craniofacial morphological characteristics between female patients with and without TMDs

|  | | **Females with no TMD**  77 (45.0%) | | | **Females with TMD**  94 (55.0%) | | | ***P*-value** |
| --- | --- | --- | --- | --- | --- | --- | --- | --- |
| **Age** (years, mean ± SD) | | 19.71 | ± | 8.05 | 23.52 | ± | 6.44 | 0.001^*^ |
| **Angle’s classification** | Angle Class I (n (%)) | 31 (40.3) | | | 36 (38.3) | | | 0.098 |
|  | Angle Class II (n (%)) | 34 (44.1) | | | 31 (33.0) | | |  |
|  | Angle Class III (n (%)) | 12 (15.6) | | | 27 (28.7) | | |  |
| **Craniofacial morphological parameters** (mean ± SD) | |  |  |  |  |  |  |  |
| Saddle angle (°) | | 123.09 | ± | 4.79 | 122.60 | ± | 5.35 | 0.416 |
| Articular angle (°) | | 152.13 | ± | 6.37 | 152.03 | ± | 7.19 | 0.925 |
| Anterior cranial base length (mm) | | 62.68 | ± | 2.79 | 63.28 | ± | 3.23 | 0.189 |
| Posterior cranial base length (mm) | | 33.57 | ± | 2.96 | 33.29 | ± | 3.12 | 0.544 |
| SNA (°) | | 81.67 | ± | 3.52 | 82.16 | ± | 3.32 | 0.349 |
| SNB (°) | | 78.62 | ± | 3.97 | 78.68 | ± | 4.35 | 0.689 |
| ANB (°) | | 3.05 | ± | 3.46 | 3.48 | ± | 3.24 | 0.582 |
| FMA (°) | | 24.44 | ± | 5.42 | 25.47 | ± | 7.20 | 0.298 |
| Gonial angle (°) | | 118.05 | ± | 6.40 | 119.54 | ± | 7.97 | 0.177 |
| Ramus height (mm) | | 44.75 | ± | 4.24 | 45.15 | ± | 5.39 | 0.603 |
| Mandibular Body length (mm) | | 69.19 | ± | 4.30 | 69.58 | ± | 4.69 | 0.576 |
| Interincisal angle (°) | | 125.32 | ± | 12.66 | 125.69 | ± | 14.93 | 0.764 |
| Cant of occlusal plane (°) | | 7.32 | ± | 4.59 | 7.37 | ± | 4.61 | 0.940 |
| Overjet (mm) | | 4.09 | ± | 2.99 | 3.66 | ± | 2.92 | 0.336 |
| Overbite (mm) | | 2.73 | ± | 2.06 | 2.57 | ± | 2.29 | 0.417 |
| Anterior facial height (mm) | | 113.68 | ± | 7.31 | 114.92 | ± | 7.15 | 0.160 |
| Posterior facial height (mm) | | 75.97 | ± | 5.63 | 76.00 | ± | 6.58 | 0.971 |
| Wits appraisal (mm) | | -0.10 | ± | 4.63 | 0.25 | ± | 4.80 | 0.558 |

Independent samples *t*-test and Mann–Whitney U-test were used. ^*^ *P* < 0.05.

TMD: temporomandibular disorder, SD: standard deviation.

**Supplement Table 2**. Comparison of demographic, occlusal, and craniofacial morphological characteristics between juvenile patients (aged under 18 years) with and without TMDs

|  | | **Juveniles with no TMD**  60 (66.7%) | | | **Juveniles with TMD**  30 (33.3%) | | | ***P*-value** |
| --- | --- | --- | --- | --- | --- | --- | --- | --- |
| **Age** (years, mean ± SD) | | 13.83 | ± | 1.81 | 15.25 | ± | 1.83 | 0.001^*^ |
| **Gender** | Male (n (%)) | 21 (35.0) | | | 13 (43.3) | | | 0.442 |
|  | Female (n (%)) | 39 (65.0) | | | 17 (56.7) | | |  |
| **Angle’s classification** | Angle Class I (n (%)) | 23 (38.3) | | | 11 (36.7) | | | 0.103 |
|  | Angle Class II (n (%)) | 28 (46.7) | | | 9 (30.0) | | |  |
|  | Angle Class III (n (%)) | 9 (15.0) | | | 10 (33.3) | | |  |
| **Craniofacial morphological parameters** (mean ± SD) | |  |  |  |  |  |  |  |
| Saddle angle (°) | | 122.75 | ± | 4.28 | 122.88 | ± | 5.01 | 0.911 |
| Articular angle (°) | | 151.10 | ± | 6.11 | 150.39 | ± | 7.74 | 0.637 |
| Anterior cranial base length (mm) | | 63.51 | ± | 3.81 | 62.73 | ± | 3.24 | 0.317 |
| Posterior cranial base length (mm) | | 34.41 | ± | 2.88 | 34.37 | ± | 3.61 | 0.946 |
| SNA (°) | | 82.26 | ± | 3.16 | 82.43 | ± | 3.18 | 0.620 |
| SNB (°) | | 78.79 | ± | 3.97 | 79.11 | ± | 4.17 | 0.723 |
| ANB (°) | | 3.47 | ± | 3.21 | 3.31 | ± | 2.90 | 0.891 |
| FMA (°) | | 24.47 | ± | 5.12 | 25.68 | ± | 7.49 | 0.433 |
| Gonial angle (°) | | 119.09 | ± | 6.68 | 120.96 | ± | 9.24 | 0.328 |
| Ramus height (mm) | | 45.02 | ± | 4.80 | 45.27 | ± | 5.44 | 0.824 |
| Mandibular Body length (mm) | | 69.21 | ± | 5.09 | 69.43 | ± | 5.62 | 0.962 |
| Interincisal angle (°) | | 123.93 | ± | 14.22 | 122.64 | ± | 11.81 | 0.647 |
| Cant of occlusal plane (°) | | 7.42 | ± | 4.27 | 8.17 | ± | 4.45 | 0.443 |
| Overjet (mm) | | 4.90 | ± | 3.58 | 3.65 | ± | 3.21 | 0.008^*^ |
| Overbite (mm) | | 3.22 | ± | 1.81 | 2.35 | ± | 2.06 | 0.042^*^ |
| Anterior facial height (mm) | | 114.44 | ± | 7.91 | 115.63 | ± | 8.88 | 0.522 |
| Posterior facial height (mm) | | 76.85 | ± | 6.27 | 76.85 | ± | 7.93 | 0.997 |
| Wits appraisal (mm) | | 0.35 | ± | 4.94 | -0.65 | ± | 4.96 | 0.535 |

Independent samples *t*-test, Mann–Whitney U-test, and chi-squared test were used. ^*^ *P* < 0.05.

TMD: temporomandibular disorder, SD: standard deviation.
